# The Oration of the Medical Society of London

**Published:** 1897-05-29

**Authors:** 


### THE ORATION OF THE MEDICAL SOCIETY OF LONDON.

The conversazione of the Medical Society, held on May 17th, was largely attended, and the oration was delivered by Mr. Edmund Owen, F.R.C.S., senior surgeon to St. Mary's Hospital. After tracing out the origin of the society in May, 1773, he worked on to August 10th of that year, when the sixth meeting was held. At this time, he said, the primary cleavage of the society into three parts was established by the appointment of three standing committees of reference —one of three physicians, one of three surgeons, and one of three apothecaries; and at the same time, as applications for membership were coming in at a great pace, the society resolved that for the present the number of its members should be limited to 30 physicians, 30 surgeons, and 30 general practitioners, or, in the language of the time, apothecaries.

The orator remarked that it could scarcely be a matter of surprise, though it might be of regret, if, with the vast increase of information which had taken place in the profession—without any proportionate increase of mental vigour and power of endurance—secondary and tertiary cleavages had since appeared. Thus every region, organ, gland, and tissue in the body had in turn been "specialised," from epiblast to hypablast, and back again. But this process had surely now gone far enough—some would think too far. For it frequently happened that the practitioner who was cultivating his little allotment of epiblast, for instance, deemed himself unable or unwise to look through a mere basement-membrane to see what morbid changes might, by chance, be taking place in the adjacent but not less important plot which his neighbour was cultivating in the mesoblast. So it had come about that one practitioner had been daily and diligently dealing with a tuberculous patch in a larynx, and refusing all the while to recognise the fact that his trusting and hopeful patient was dying of pulmonary consumption; and that another practitioner had been lumbering up the weak back of a growing girl with what he was pleased to call a "spinal support," when all that the girl needed was sunlight, fresh air, exercise, and freedom.

Mr. Owen was of opinion that every one of those present must feel that he was not erring upon the side of exaggeration in making these remarks; and if such things had happened in the past, what was going to happen in the future? A very large proportion of special practitioners of the present time were fortunately under the inhibitory influence of broad principles which had been acquired in the useful and essential preliminary work of general medicine, general surgery, or general practice. But henceforth, it seemed, the youthful practitioner was to embark in the cultivation of some speciality almost before the ink on his diploma was dry. One heard it urged that the public liked and desired this sort of thing. He could not stay to discuss that statement, or even to deny its truth. But supposing that the public did like it, was that a reason why medical men should stand quietly by whilst their profession, as practised by Fothergill and Lettsom, was brought down to the level of persons who nourish their children upon patent foods and themselves upon quack medicines?

